# Allelic Expression Changes in Medaka (*Oryzias latipes*) Hybrids between Inbred Strains Derived from Genetically Distant Populations

**DOI:** 10.1371/journal.pone.0036875

**Published:** 2012-05-10

**Authors:** Yasuhiko Murata, Shoji Oda, Hiroshi Mitani

**Affiliations:** Department of Integrated Biosciences, Graduate School of Frontier Sciences, The University of Tokyo, Kashiwa, Chiba, Japan; North Carolina State University, United States of America

## Abstract

Variations in allele expressions between genetically distant populations are one of the most important factors which affects their morphological and physiological variations. These variations are caused by natural mutations accumulated in their habitats. It has been reported that allelic expression differences in the hybrids of genetically distant populations are different from parental strains. In that case, there is a possibility that allelic expression changes lead to novel phenotypes in hybrids. Based on genomic information of the genetically distant populations, quantification and comparison of allelic expression changes make importance of regulatory sequences (cis-acting factors) or upstream regulatory factors (trans-acting modulators) for these changes clearer. In this study, we focused on two Medaka inbred strains, Hd-rR and HNI, derived from genetically distant populations and their hybrids. They are highly polymorphic and we can utilize whole-genome information. To analyze allelic expression changes, we established a method to quantify and compare allele-specific expressions of 11 genes between the parental strains and their reciprocal hybrids. In intestines of reciprocal hybrids, allelic expression was either similar or different in comparison with the parental strains. Total expressions in Hd-rR and HNI were tissue-dependent in the case of *HPRT1*, with high up-regulation of Hd-rR allele expression in liver. The proportion of genes with differential allelic expression in Medaka hybrids seems to be the same as that in other animals, despite the high SNP rate in the genomes of the two inbred strains. It is suggested that each tissue of the strain difference in trans-acting modulators is more important than polymorphisms in cis-regulatory sequences in producing the allelic expression changes in reciprocal hybrids.

## Introduction

Variations in allele expressions between genetically distant populations are one of the most important factor which affects to their morphological and physiological variations. These variations are caused by natural mutations accumulated in their habitats. Over the past years, many studies have found natural hybrid zones where distant species, subspecies and races live and genetic exchange occurs. These zones are thought to be rich sources of information for evolutionary genetic studies [1]. In hybrids, mixture of alleles derived from genetically distant populations can lead to different allelic expressions from their parental populations. These differential allelic expressions are common in many species [2]–[Bibr pone.0036875-Guo1] [Bibr pone.0036875-Cowles1] [5], and can result from mutations in its regulatory sequences (cis-acting factors) or from mutations elsewhere in the genome that alter the transcriptional factors (trans-acting modulators). It has been reported that cis-acting factors dominated in yeast [6], Drosophila [7], [8] and human [9].

Medaka, *Oryzias latipes*, is a fresh-water bony fish inhabiting China, Korea and Japan. There have been many studies on phenotypic diversity and phylogenetic relationships using this species [10]–[Bibr pone.0036875-Sakaizumi1] [Bibr pone.0036875-Shima1] [13]. It also has a long history as an experimental animal and many inbred strains have been established. In particular, whole-genome sequence alignment of two medaka inbred strains, Hd-rR and HNI, which are derived from two regional populations (the southern Japanese population and the northern Japanese population) has uncovered that the genome-wide SNP rate is 3.4% and the SNP rate in coding regions is 1.8% [14]. Despite the accumulation of genetic variation, these strains can mate and produce healthy and fertile offspring. Comparative genomic analysis has suggested that such large genetic differences between the two populations are caused by higher molecular evolutionary rates based on the assumption that the two Japanese populations diverged at approximately the same time (4.0–4.7 Myr ago). However, Setiamarga et al. (2009) concluded based on a Bayesian relaxed molecular-clock analysis of whole mitogenome sequences that the divergence time was 18 Myr ago and that reproductive isolation may not evolve despite a long period of geographical isolation [15].

Based on genomic information, quantification and comparison of allelic expression changes between the tissues of Medaka inbred strains derived from genetically distant populations and their hybrids makes their process of transcriptional evolution clearer. In this study, we developed allele-specific quantification assays and found that strain- and tissue-specific transcriptional regulatory factors are important for allelic expression changes in hybrids.

## Materials and Methods

### Medaka strains and their reciprocal hybrids

Six individuals from each strain and reciprocal hybrid (inbred strains, Hd-rR, and HNI (HNI-II), the hybrid of HNI female and Hd-rR male, NdF1 and the hybrid of Hd-rR female and HNI male, dNF1) were used in our study. The HNI-II strain (No. IB176) used in this research was provided by the National BioResource Project (NBRP), MEXT, Japan. All were mature male fishes and more than three months old after hatch. All fishes were maintained at 26°C under a 14/10 h light/dark cycle. The homo- and heterozygosity of M-marker [16] locus in each individual was confirmed and the genomic sequence of the sex-determining gene DMY [17] was also checked to confirm the sex (data not shown).

### Total RNA extraction and reverse transcription

Total RNA was extracted from intestine, liver and brain of mature males using ISOGEN (Nippon Gene, Tokyo, Japan) according to the manufacturer's instructions. The RNA was subjected to genomic DNA degradation and RNA purification using an RNase-free DNase kit and an RNeasy MinElute Cleanup kit (Qiagen, Tokyo, Japan), respectively, according to the manufacturer's protocols. First-strand cDNA was synthesized using the ReverTra Ace-α system (Toyobo, Osaka, Japan) from 500 ng of total RNA following the manufacturer's instructions. To evaluate allele-specific expression, quantitative real-time PCR (qRT-PCR) was conducted using SYBR Premix Ex Taq (TaKaRa, Shiga, Japan) with a Smart Cycler II system (Cepheid, Sunnyvale, CA) following the manufacturer's instructions.

For screening with allele-specific primers, PCR and qRT-PCR were conducted under the following conditions. TaKaRa ExTaq DNA polymerase (TaKaRa) was used for PCR. Thermal cycling conditions consisted of an initial step at 94°C for 1 min, followed by 30 or 35 cycles of degeneration at 94°C for 30 s, annealing at 60°C for 30 s, and polymerization at 72°C for 30 s. In qRT-PCR, the thermal cycling conditions consisted of 95°C for 5 s and 60°C for 20 s until detection of fluorescence.

### Designing of primers for amplification of allele-specific transcripts

Eleven genes (mini-chromosome maintenance proteins 2 (*MCM2*), proliferating cell nuclear antigen (*PCNA*), glyceraldehyde-3-phosphate dehydrogenase (*GAPDH*), citrate synthase (*CS*), metallothionein (*MT*), cytochrome P450 family 2 subfamily J number 2 (*CYP2J2*), fructose-bisphosphate aldolase B (*ALDOB*), trypsinogen (*TRYP*), flavin-containing monooxygenase (*FMO*), hypoxanthine phosphoribosyltransferase 1 (*HPRT1*), and proteasome subunit β-type 8 (*PSMB8*)) were selected randomly from the genome database and allele-specific primers were designed.

**Figure 1:**
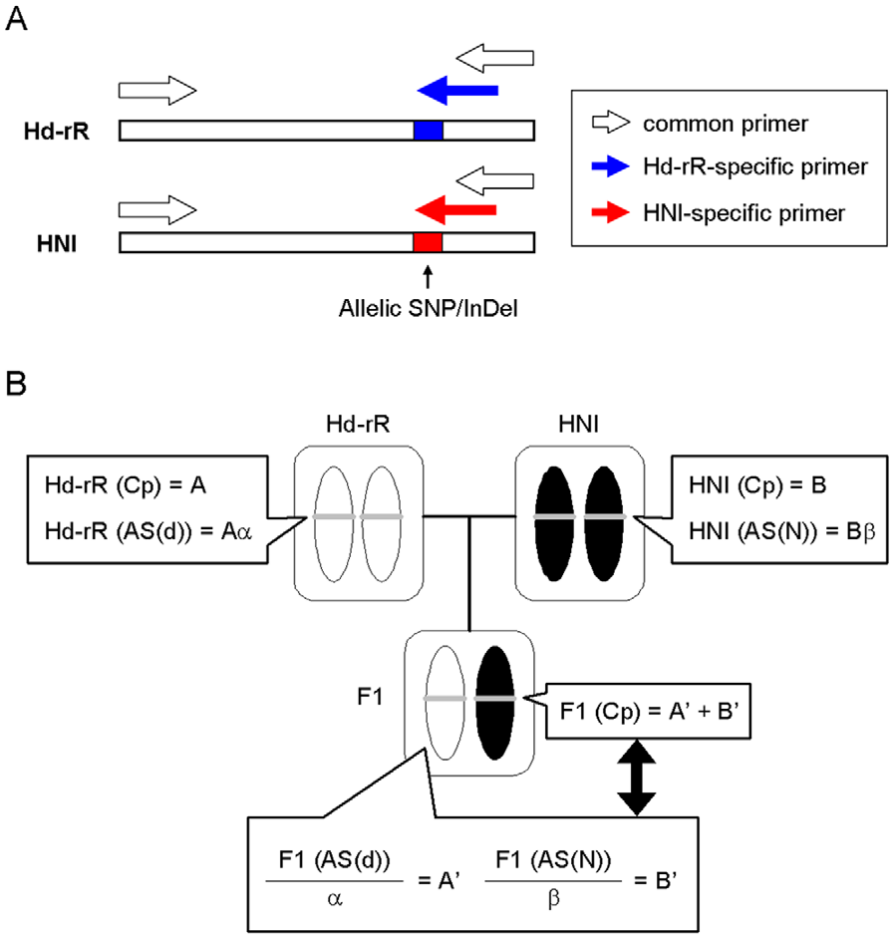
Quantification of total and allelic expression. (A) Common and allele-specific primers were designed for quantification of total and allelic expressions in a target gene. In each allele-specific primer, Single nucleotide polymorphisms (SNPs), insertions, deletions (InDel) or substitutions were placed at the 3′ ends of primers and the second base from the 3′ end of each primer was substituted to be a mismatch nucleotide for discrimination of Hd-rR and HNI transcripts. Use of the three types of primers enabled comparisons of allele-specific expression in reciprocal hybrids. (B) Allelic expression (A′ or B′) in hybrid were calculated using correction factors, α and β, for each allele-specific primer (see ‘Materials and Methods’ section). These factors were determined from ratio of total expressions (e.g. Hd-rR(Cp) and Hd-rR(AS(d))) quantified by common and allele-specific primers in each parental strain. Cp: common primer. AS(d) and AS(N): allele-specific primer for Hd-rR or HNI allele. α and β: the ratio of total expressions of common and allele-specific primers.

Three types of primers, allele-specific primers for Hd-rR and HNI alleles and common primers for consensus sequences, were designed using expressed sequence tags and genome sequences of Hd-rR and HNI strains acquired from Ensembl (http://www.ensembl.org/index.html), NBRP Medaka (http://www.shigen.nig.ac.jp/medaka/) and NIG databases (http://dolphin.lab.nig.ac.jp/medaka/) (Fig. 1A). The polymorphisms distinguishing the two inbred strains, single nucleotide polymorphisms (SNPs), insertions, deletions (in/del) or substitutions were used to ensure allele specificity. When we targeted a single nucleotide polymorphism (SNP), it was designed to be placed at the 3′-end of a primer and deliberate mismatch nucleotides were inserted at the second base from the 3′ end for more effective allele discrimination [18]. Inserted mismatch nucleotides markedly inhibit the extension reaction of DNA polymerase on non-targeted allele-specific transcripts [19], [20]. All primers were designed using the Primer3Plus program (http://www.bioinformatics.nl/cgi-bin/primer3plus/primer3plus.cgi) with the qRT-PCR condition and the uniqueness of sequences was checked using the In-Silico PCR program at the University of California, Santa Cruz (UCSC) Genome Browser (http://genome.ucsc.edu/) or BLAST program at Ensembl using all the designed primers. All primer sequences and accession numbers of the genes are shown in Table S1.

### Quantitative analysis of allele-specific expression

Total expression which is sum of the two allele expression in each parental strain or F1 hybrid was quantified by common primer designed at consensus sequences and normalized values relative to abundance of *β-actin* transcripts were calculated according to the manufacture's procedures (Takara). The abundance of target gene and *β-actin* transcripts was calculated from a slope and an intercept of Hd-rR cDNA standard curve and Ct (cycle threshold) values. Allelic expressions which are relative to abundance of *β-actin* transcripts were also calculated from abundance of target gene transcripts using Ct values quantified by allele-specific primer. However, comparison of the two allele expressions in F1 hybrid with different allele-specific primers was not appropriate because all the Hd-rR and HNI allele-specific and common primers for target genes show different amplification efficiency. To compare total and allelic expressions, correction factors, α and β, for each allele-specific primer were determined (Fig. 1B). We show an example that calculation of allelic expression using a correction factor α for the different amplification efficiency between common and Hd-rR allele-specific primers.
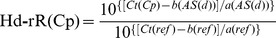
(1)

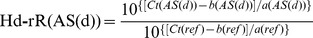
(2)Equation **1** and **2** show total expressions in Hd-rR quantified by common and Hd-rR allele-specific primers. Cp is common primer; AS(d) is Hd-rR allele-specific primer; d is Hd-rR; Hd-rR(Cp) and Hd-rR(AS(d)) are total expressions in Hd-rR quantified by common and Hd-rR allele-specific primers; Ct(Cp), Ct(AS(d)) and Ct(ref) are Ct values in Hd-rR quantified by common, Hd-rR allele-specific and *β-actin* primers; a(AS(d)) and a(ref) are slopes calculated from Hd-rR cDNA dilution series using Hd-rR allele-specific primer and *β-actin* primer; b(AS(d)) and b(ref) are intercepts calculated from Hd-rR cDNA dilution series using Hd-rR allele-specific and *β-actin* primer.
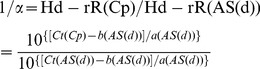
(3)

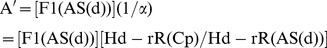
(4)Equation **3** shows calculation of the correction factor α and equation **4** shows a calculation of Hd-rR allele expression in F1 hybrid. F1(AS(d)) is Hd-rR allele-expression in F1 hybrid quantified by Hd-rR allele-specific primer; A′ is Hd-rR allele expression after correction of different amplification efficiencies. Calculation of correction factor β and HNI allele expression in F1 hybrid were conducted in the same way.

### Statistical analysis

Six individuals were used and the tables and figures were constructed from the data of three independent experiments for each individual. Student's *t*-tests and Bonferroni corrections were used to compare total and allelic expressions quantified by common and allele-specific primers between parents and reciprocal hybrids. All results are expressed as the mean ± standard deviation (SD). Statistical significance is described in figure legends.

## Results

### Screening of allele-specific primers

**Figure 2:**
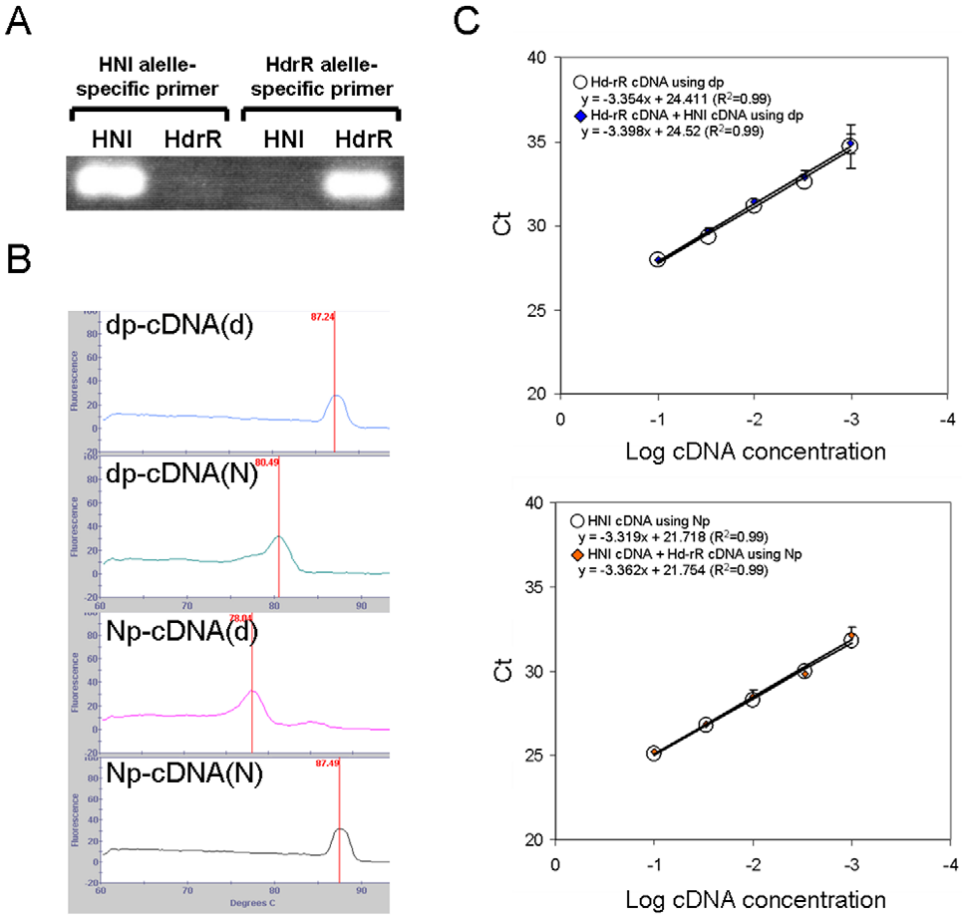
Allele-specificity and quantitative reproducibility of allele-specific primers. (A) Hd-rR or HNI allele transcripts of target gene were amplified by allele-specific primers, respectively. (B) Target gene transcripts amplified by allele-specific primers showed a major single peak in qRT-PCR experiments. (C) Ct values obtained from a set of serial dilution cDNA and from mixed cDNA of parental strains at five different ratios (1∶1, 1∶3, 1∶10, 1∶30, 1∶100) showed that each allele-specific primer pair have allele specificity and quantitative reproducibility. Hd-rR allele-specific primer for *HPRT1* (upper), HNI allele-specific primer for *HPRT1* (lower). dp: Hd-rR allele-specific primer, Np: HNI allele-specific primer.

To validate allele specificity of the designed allele-specific primers for the target gene, PCR discrimination was conducted and PCR products amplified using cDNA synthesized from total RNA of Hd-rR and HNI intestines showed predicted sizes (Fig. 2A). Next, the primers that showed at least four-fold difference between Ct (cycle threshold) values in Hd-rR and HNI cDNAs were screened by qRT-PCR (Fig. 2B). To demonstrate the allele specificity and quantitative reproducibility of the screened primers, we prepared a set of serial dilution (1/10, 1/30, 1/100, 1/300, 1/1000) and mixed cDNAs of parental strains at five different ratios for each allele-specific primer pair (Hd-rR cDNA∶HNI cDNA or HNI cDNA∶Hd-rR cDNA; 1∶1, 1∶3, 1∶10, 1∶30, 1∶100). All Ct values obtained from serial dilution of each cDNA and mixed cDNAs using each allele-specific primer pair were plotted and the two standard curves were compared. Each allele-specific primer pair was selected when the difference between the intercepts of the two standard curves was under 1 in replicate experiments. All the standard curves obtained from 11 allele-specific primer pairs using the mixed cDNAs of parental strains at different ratios showed almost same with serial dilution of each cDNA (Fig. 2C and Fig. S1). To further validation, we designed another mixed cDNAs of parental strains at three known expression ratios (HNI allele expression∶Hd-rR allele expression; 1∶3, 1∶1 and 3∶1) for 11 genes and conducted quantification of each allele expression. All genes showed nearly expression ratio with known expression ratios (Table S2).

### Comparing total and allelic expression between the parental strains and the F1 hybrids

**Figure 3:**
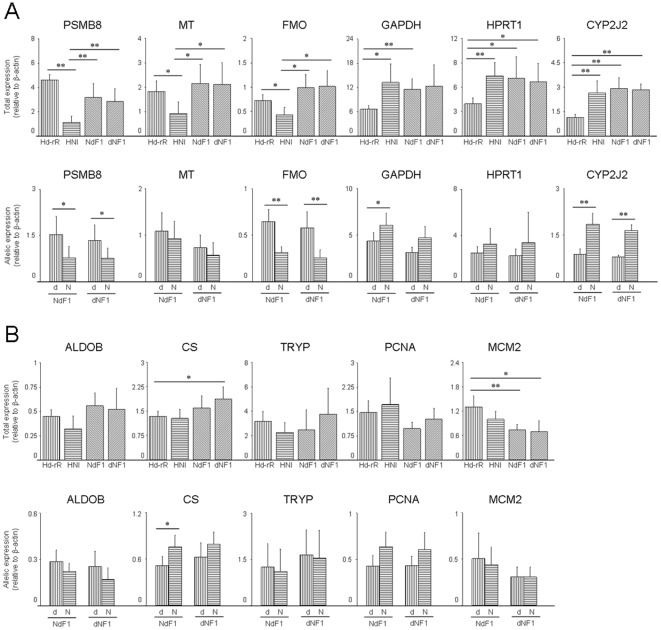
*PSMB8, MT, GAPDH and HPRT1* in reciprocal hybrids showed decreasing total expression differences between parental strains. (A) Six (*PSMB8*, *MT*, *FMO*, *CYP2J2*, *HPRT1* and *GAPDH*) out of 11 genes showed significantly different total expressions between parental strains (upper). Three (PSMB8, FMO, CYP2J2) out of the 6 genes showed significantly different allelic expressions between Hd-rR and HNI allele in reciprocal hybrids (lower). (B) Five out of 11 genes did not show significantly different total expressions between parental strains (upper) and allelic expressions in reciprocal hybrids (lower). All expressions were normalized by *β-actin* expressions. Data is presented as mean ± SD, n = 6. * *P*<0.05, ** *P*<0.01.

We analyzed the total and allelic expression of randomly selected 11 genes in the intestines of parental strains and the reciprocal hybrids (NdF1 and dNF1) using common and allele-specific primers (Fig. 3 and Table S3). Six (*PSMB8*, *MT*, *FMO*, *CYP2J2*, *HPRT1* and *GAPDH*) out of 11 genes showed significant total expression differences between parental strains (Fig. 3A). Five of these excluding *GAPDH* also showed significantly higher total expression in reciprocal hybrids than in one of the parental strains which showed lower total expression. On the other hand, *GAPDH* was not significantly higher total expression in dNF1 than in one of the parental strains. The other five genes (*ALDOB*, *TRYP*, *MCM2*, *CS*, *PCNA*) did not show significantly different total expression between parental strains (Fig. 3B).

In comparison with total expressions between parental strains, reciprocal hybrids showed similar increases in intestinal expression from both alleles of the *FMO* and *CYP2J2*, and the ratios of Hd-rR and HNI allele expressions in reciprocal hybrids (d/N ratio) were as expected from the total expression ratios of the parental strains (Hd-rR/HNI ratio) (Fig. 3A and Table S3). Interestingly, *MT* and *HPRT1* in reciprocal hybrids did not show significant allelic expression differences and the d/N ratio of these genes were lower than expected Hd-rR/HNI ratios (Fig. 3A and Table S3). *PSMB8* in reciprocal hybrids and *GAPDH* in NdF1 showed significant allelic expression differences, and *GAPDH* in dNF1 did not show significant allelic expression difference. However, the d/N ratio of both genes in reciprocal hybrids were lower than expected Hd-rR/HNI ratios (Fig. 3A and Table S3). For all genes examined, the total and sum of the allele expressions in reciprocal hybrids quantified by common primers and allele-specific primers were consistent (Fig. S2 and Table S4). Moreover, the d/N ratio was not significantly different between reciprocal hybrids (Fig. S3).

### Comparing total and allelic expression between three tissues

**Figure 4:**
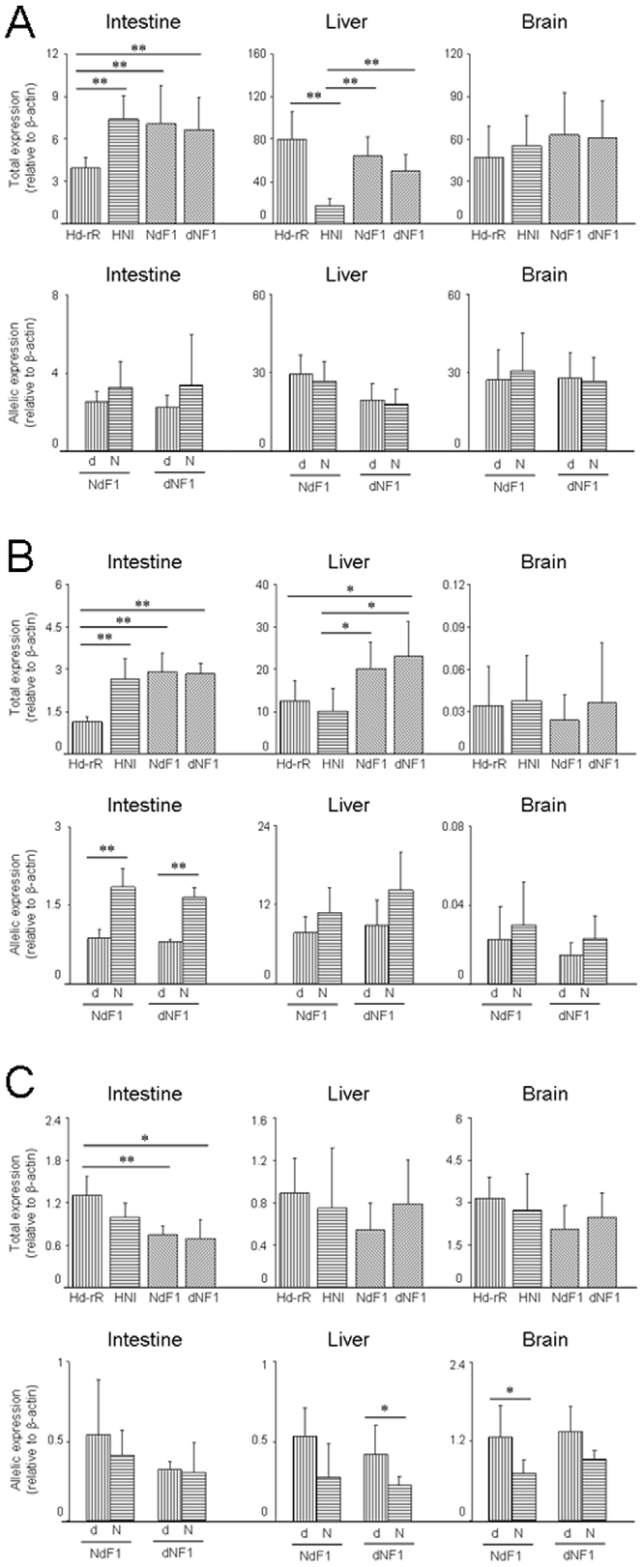
*HPRT1* total expression difference between liver of parental strains showed opposite difference in intestine and decreasing allelic expression difference in reciprocal hybrids. Total and allelic expressions of three genes ((A) *HPRT1*, (B) *CYP2J2* and (C) *MCM2*) in intestine, liver and brain of parental strains and reciprocal hybrids were quantified by common and allele-specific primers. All expressions were normalized by *β-actin* expressions. Data is presented as mean ± SD, n = 6. * *P*<0.05, ** *P*<0.01.

To examine the differences between total expression in other tissues (brain and liver) of parental strains and reciprocal hybrids, we chose *HPRT1*, *CYP2J2* and *MCM2* which showed significantly different or not different total expression between parental intestines (Fig. 4 and Table S5). Interestingly, in intestine, total expression of *HPRT1* in HNI was significantly higher than that in Hd-rR, but, in liver, the total expression in Hd-rR was significantly higher about 4-fold than that in HNI. Therefore Hd-rR/HNI ratio of *HPRT1* in liver was opposite to the observed Hd-rR/HNI ratio in intestine (Fig. 4A and Table S5). The total amount of *HPRT1* transcripts in reciprocal hybrids was the same as that in the parental strain, but the d/N ratios were smaller than expected from Hd-rR/HNI ratio. This decrease in d/N ratios was caused by up-regulation of the HNI allele and down-regulation of the Hd-rR allele. The d/N ratios of *CYP2J2* in all tissues of reciprocal hybrids were as expected from Hd-rR/HNI ratio and higher in liver than in intestine and brain (Fig. 4B and Table S5). Although, in three tissues, significant total expression differences were not found in *MCM2*, significant allelic expression differences were found in liver of dNF1 and brain of NdF1, (Fig. 4C). For all genes examined, total and allelic expressions in reciprocal hybrids quantified by common primers and allele-specific primers were consistent (Fig. S4 and Table S6). Moreover, the d/N ratio was not significantly different between reciprocal hybrids (Fig. S5).

## Discussion

In geographically isolated populations, it seems very possible that significant accumulation of natural mutations could cause modification of gene expression suitable for adaptation to their environment. Medaka are highly diverse showing geographic variations, and experimental inbred strains from distant populations are available [12]. The rate of SNPs in the genome (3.4%) and coding regions (1.8%), and the average *K_A_/K_S_* (0.43) are very high among vertebrate species [14], so it is a useful model in which to perform quantification of allele-specific expression among wild populations and their hybrids. Genes with non-synonymous SNPs showing high divergence in humans are polymorphic in the same or the close sites in Medaka, and some show signals of positive selection [13], [21]. Therefore, we selected two inbred strains derived from northern and southern Japanese populations with draft genome information.

Our results indicated that up- or down-regulation of allele-specific expression of 4 (*PSMB8*, *MT*, *GAPDH* and *HPRT1*) out of 11 genes in intestines of F1 hybrids causes the disappearance of allelic expression differences though total expression differences were observed between parental strains. When total expression is different among strains, it is thought that the mutations accumulate in the regulatory region of the gene and/or transcription factors have different activities. If the total expression differences are determined by polymorphisms only in the regulatory region of a gene, allelic expression should be constant in reciprocal hybrids, and the total amount of allelic expression should be near the average of each total expression in parental strains with a similar d/N ratio to Hd-rR/HNI ratio. However, none of the genes that showed significant difference in total expressions between parental strains showed such results. Taken together, our results indicated that changes in the transcriptional factors involved in both activation or inhibition of allelic expression are more important in reciprocal hybrids. Interestingly, *HPRT1* in liver of parental strains showed the opposite total expression differences to that in intestine (Fig. 4A and Table S5). However, up- or down-regulation of allele-specific expression in each reciprocal hybrid also causes the disappearance of total expression differences between parental strains. Such regulation may be constant among tissues regardless of extent of total expression differences between parental strains.

We found that the total expressions of *HPRT1* in liver and brain of any parental strain were higher than that in intestine (Fig. 4A). The expression of *HPRT1* from Hd-rR allele was high in parents, but largely suppressed in reciprocal hybrids (Fig. 4 and Table S5). In the coding region of *HPRT1*, two out of seven SNPs were non-synonymous substitutions (data not shown) so that the *HPRT1* proteins produced from the Hd-rR allele may have a functional difference or a transcriptional suppressor is possibly produced by some HNI allele(s) in liver and brain of reciprocal hybrids.

In this study, genes did not show significantly different allelic expression and d/N ratios between reciprocal hybrids (Fig. S2, S3, S4, S5). *DNMT3L*, a key regulator of genomic imprinting, is not coded in Zebrafish, Fugu genomes [22]–[Bibr pone.0036875-Hata1] [24]. We searched *DNMT3L* in Medaka genome database using BLAST program, but orthologous gene was not found. There is a possibility that parent-of-origin-specific expression control by *DNMT3L* is not occurred in Medaka. In fish including Medaka, it has been well known that sex hormone induce sex reversal [25]. This report also supports the possibility described above.

Our results suggest that a considerable number of genes show differential allelic expressions in terms of their expression in reciprocal hybrids. However, the number of genes examined in this study is small; they were selected based on the presence of allelic sequence polymorphisms and otherwise represent a random set of genes. A study in mouse hybrids [4] showed that ∼10% of genes analyzed (7 out of 69 genes) showed significant allelic expression difference ranging from 1.5-fold to 4-fold. Yan et al. (2002) showed that 6 out of 13 human genes have allelic expression with a 1.3- to 4.3-fold difference [2]. Differential allelic expression has been estimated to affect 20–50% of genes in humans [5], [26]. The proportion of genes with allelic expression differences in Medaka reciprocal hybrids seems to be the same as that in other animals, despite the very high SNP rate. It is expected that genome-wide detection of allele-specific expression using high-throughput sequencing technologies will reveal the Hd-rR- and the HNI allele-specific transcriptional mechanisms in the near future.

This work also demonstrates that Medaka inbred strains and their hybrids are suitable for studying allelic expression because two alleles are transcribed under the same conditions and equally controlled by the hybrid genome. Our method established here should shed light on the nature of allelic expression changes among hybrids of the other diploid organisms.

## Supporting Information

Figure S1 Ct values obtained from Hd-rR allele-specific primers for 10 genes excluding *HPRT1* (upper) and HNI allele-specific primers for 10 genes excluding *HPRT1* (lower) using serial dilution cDNA and mixed cDNA of parental strains were plotted, respectively. Ct values from a set of serial dilution cDNA and from mixed cDNA of parental strains at five different ratios (1∶1, 1∶3, 1∶10, 1∶30, 1∶100) showed allele specificity and quantitative reproducibility of each allele-specific primer. Data is presented as mean ± SD, n = 3. dp: Hd-rR allele-specific primer, Np: HNI allele-specific primer.
(TIF)

Figure S2 Comparison of total expressions and sum of the two allele expressions in reciprocal hybrids. Total expressions and sum of the two allele expressions of 11 genes in intestines of NdF1 (A) and dNF1 (B) were quantified by common primers and the two allele-specific primers respectively. All expressions were normalized by *β-actin* expressions and were plotted on a logarithmic scale.
(TIF)

Figure S3 Comparison of the d/N ratios in 11 genes between reciprocal hybrids. All d/N ratios were plotted on a logarithmic scale.
(TIF)

Figure S4 Comparison of total expressions and sum of the two allele expressions of three genes (*MCM2*, *HPRT1* and *CYP2J2*) in intestine, liver and brain of reciprocal hybrids. Total expression and sum of the two allele expressions of 11 genes in intestines of NdF1 (A) and dNF1 (B) were quantified by common primers and the two allele-specific primers, respectively. All expressions were normalized by *β-actin* expressions and were plotted on a logarithmic scale.
(TIF)

Figure S5 Comparison of the d/N ratios of 3 genes (*MCM2*, *HPRT1* and *CYP2J2*) in intestine, liver and brain of reciprocal hybrids. All d/N ratio were plotted on a logarithmic scale.
(TIF)

Table S1 All common and allele-specific primer sequences and locations for the 11 genes. Small letters in primer sequences represent mismatch nucleotide.
(TIF)

Table S2 Hd-rR and HNI allele expression ratio of 11 genes were quantified using a mixed cDNA of parental strains at known ratio (HNI allele expression∶Hd-rR allele expression; 1∶3, 1∶1 and 3∶1). All expressions were normalized by *β-actin* expressions. Data is presented as mean ± SD, n = 3.
(TIF)

Table S3 Total expressions of 11 genes in parental strains and allelic expression of 11 genes in intestines of reciprocal hybrids quantified by common and allele-specific primers. Hd-rR/HNI ratio: (total expression in Hd-rR)/(total expression in HNI), d/N ratio: (Hd-rR allele expression)/(HNI allele expression). NdF1: hybrid of female HNI and male Hd-rR, dNF1: hybrid of female Hd-rR and male HNI. All expressions were normalized by *β-actin* expressions. Data is presented as mean ± SD, n = 6. *P<0.05, **P<0.01.
(TIF)

Table S4 Total expression of 11 genes and sum of the two allele expressions of 11 genes in intestines of reciprocal hybrids quantified by common and allele-specific primers. All expressions were normalized by *β-actin* expressions. Data is presented as mean ± SD, n = 6.
(TIF)

Table S5 Total expressions of 3 genes (*HPRT1*, *CYP2J2* and *MCM2*) in parental strains and allelic expression of 3 genes in three tissues (intestine, liver and brain) of reciprocal hybrids quantified by common and allele-specific primers. Hd-rR/HNI ratio: (total expression in Hd-rR)/(total expression in HNI), d/N ratio: (Hd-rR allele expression)/(HNI allele expression). NdF1: hybrid of female HNI and male Hd-rR, dNF1: hybrid of female Hd-rR and male HNI. All expressions were normalized by *β-actin* expressions. Data is presented mean ± SD, n = 6 *P<0.05, **P<0.01.
(TIF)

Table S6 Total expression of 3 genes (*HPRT1*, *CYP2J2* and *MCM2*) and sum of the two allele expressions of 3 genes in three tissues (intestine, liver and brain) of reciprocal hybrids quantified by common and allele-specific primers. All expressions were normalized by *β-actin* expressions. Data is presented as mean ± SD, n = 6.
(TIF)
