# Supplementary figures and images for: Allelic Expression Changes in Medaka (Oryzias latipes) Hybrids between Inbred Strains Derived from Genetically Distant Populations
Source: PLoS One. 2012 May 10;7(5):e36875. doi: 10.1371/journal.pone.0036875 (PMC3349633; doi:10.1371/journal.pone.0036875)

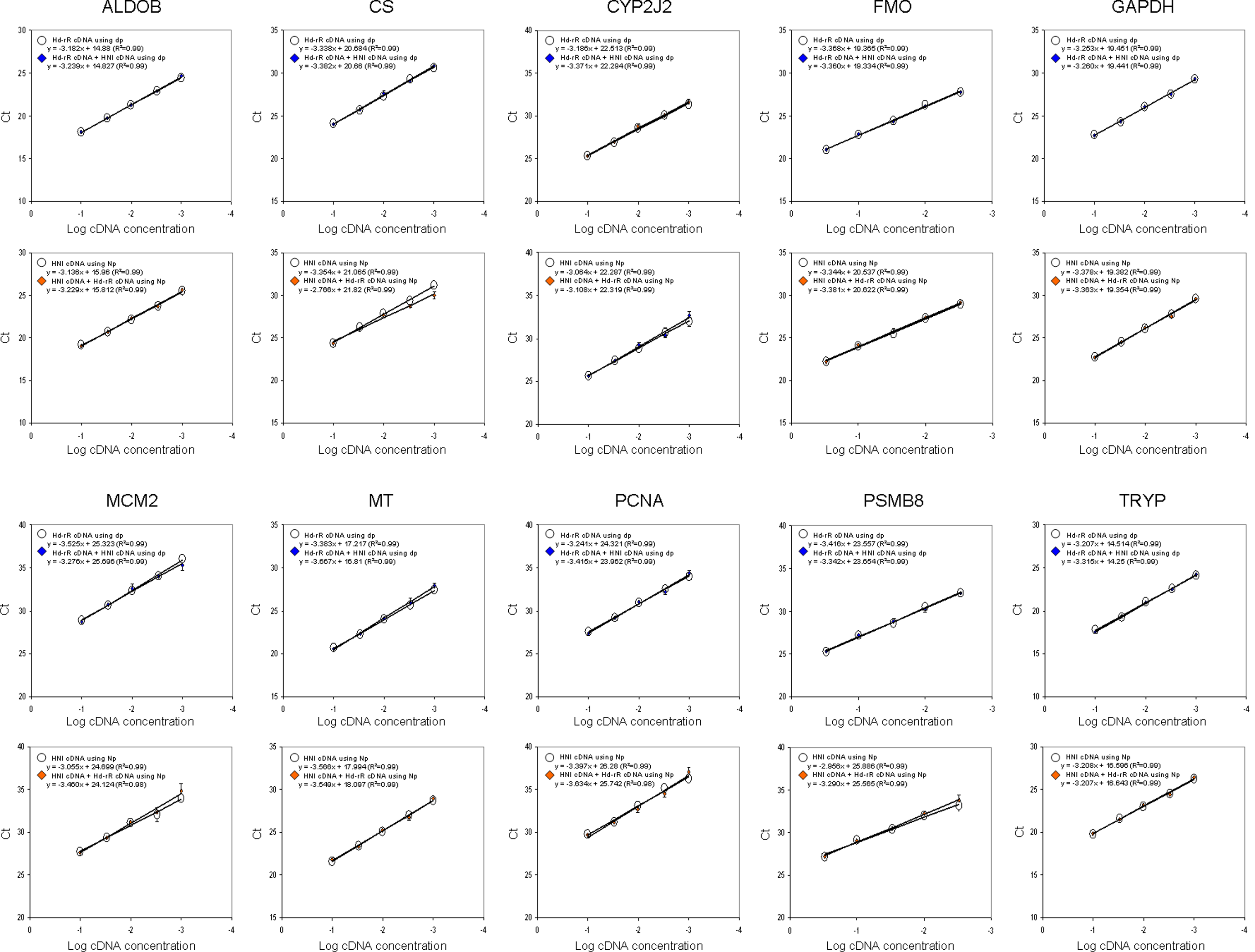

Supplement: Figure S1 — Ct values obtained from Hd-rR allele-specific primers for 10 genes excluding HPRT1 (upper) and HNI allele-specific primers for 10 genes excluding HPRT1 (lower) using serial dilution cDNA and mixed cDNA of parental strains were plotted, respectively. Ct values from a set of serial dilution cDNA and from mixed cDNA of parental strains at five different ratios (1∶1, 1∶3, 1∶10, 1∶30, 1∶100) showed allele specificity and quantitative reproducibility of each allele-specific primer. Data is presented as mean ± SD, n = 3. dp: Hd-rR allele-specific primer, Np: HNI allele-specific primer. (TIF) [file pone.0036875.s001.tif]

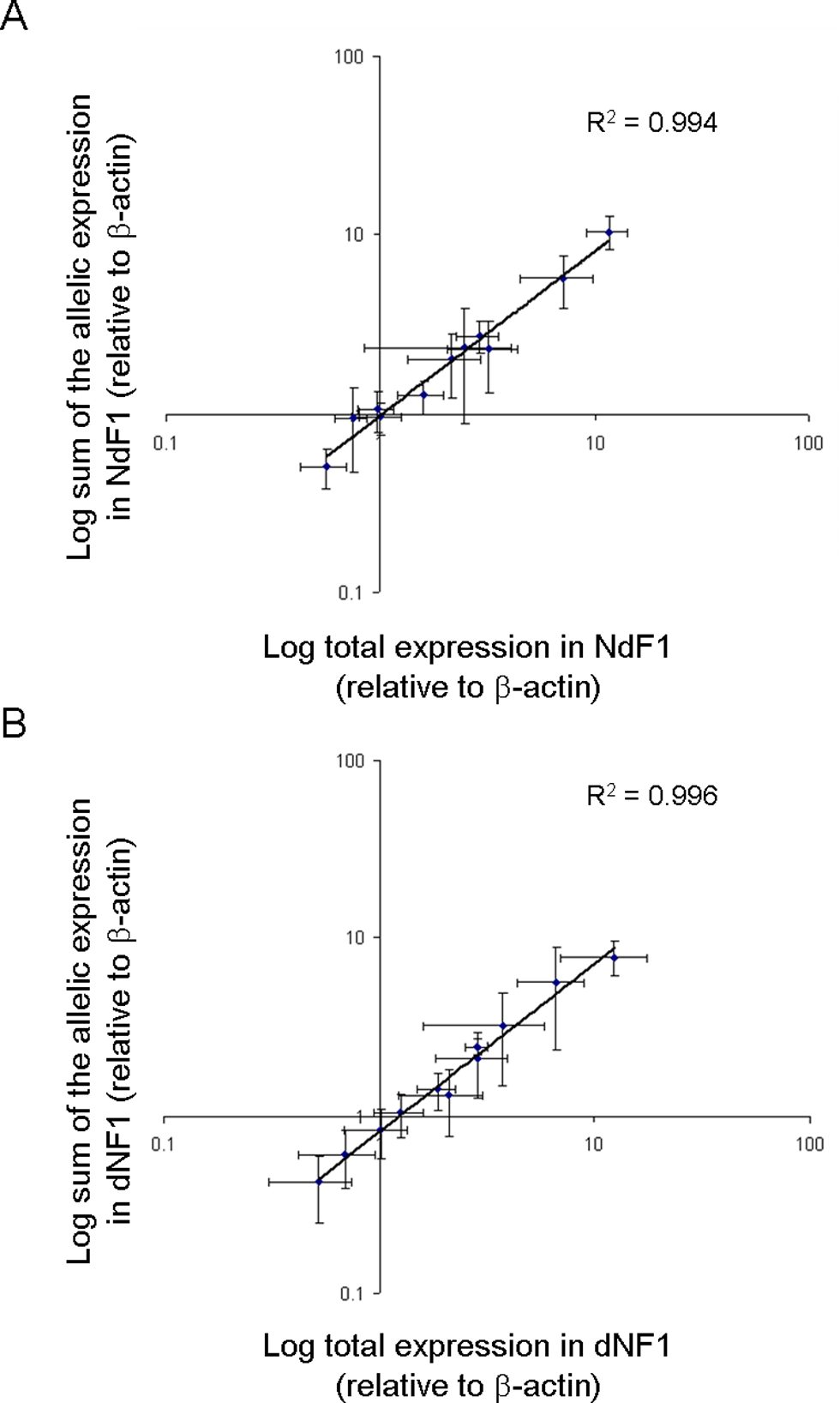

Supplement: Figure S2 — Comparison of total expressions and sum of the two allele expressions in reciprocal hybrids. Total expressions and sum of the two allele expressions of 11 genes in intestines of NdF1 (A) and dNF1 (B) were quantified by common primers and the two allele-specific primers respectively. All expressions were normalized by β-actin expressions and were plotted on a logarithmic scale. (TIF) [file pone.0036875.s002.tif]

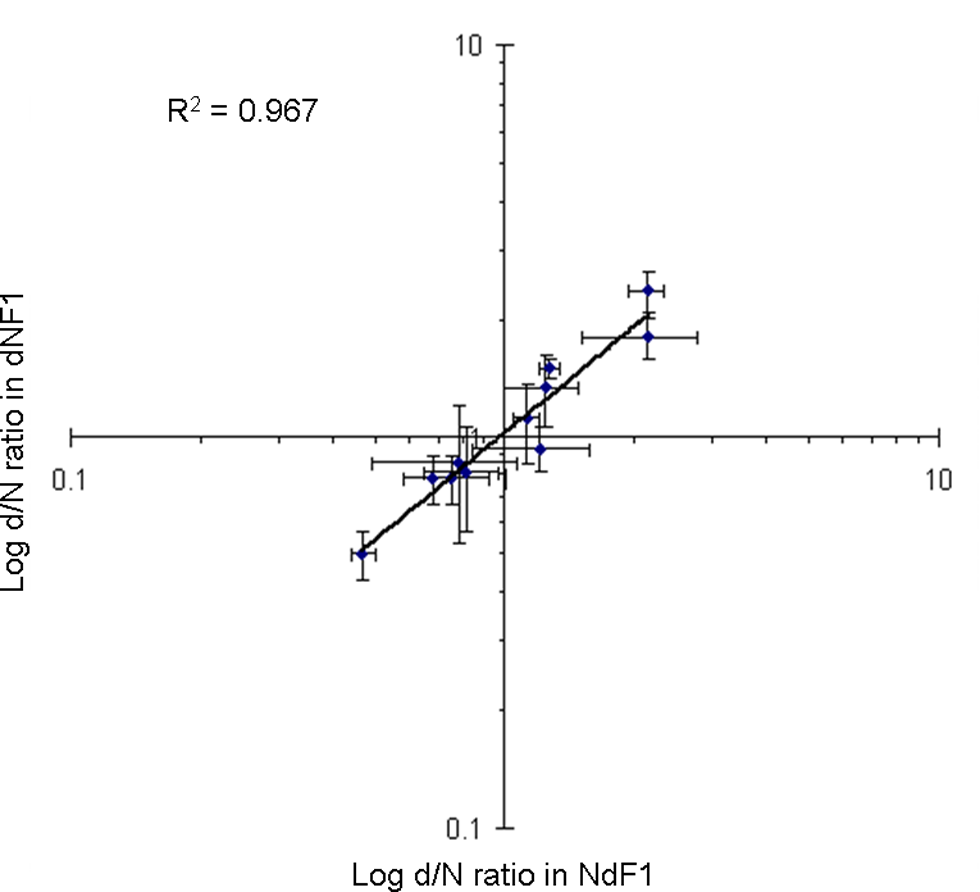

Supplement: Figure S3 — Comparison of the d/N ratios in 11 genes between reciprocal hybrids. All d/N ratios were plotted on a logarithmic scale. (TIF) [file pone.0036875.s003.tif]

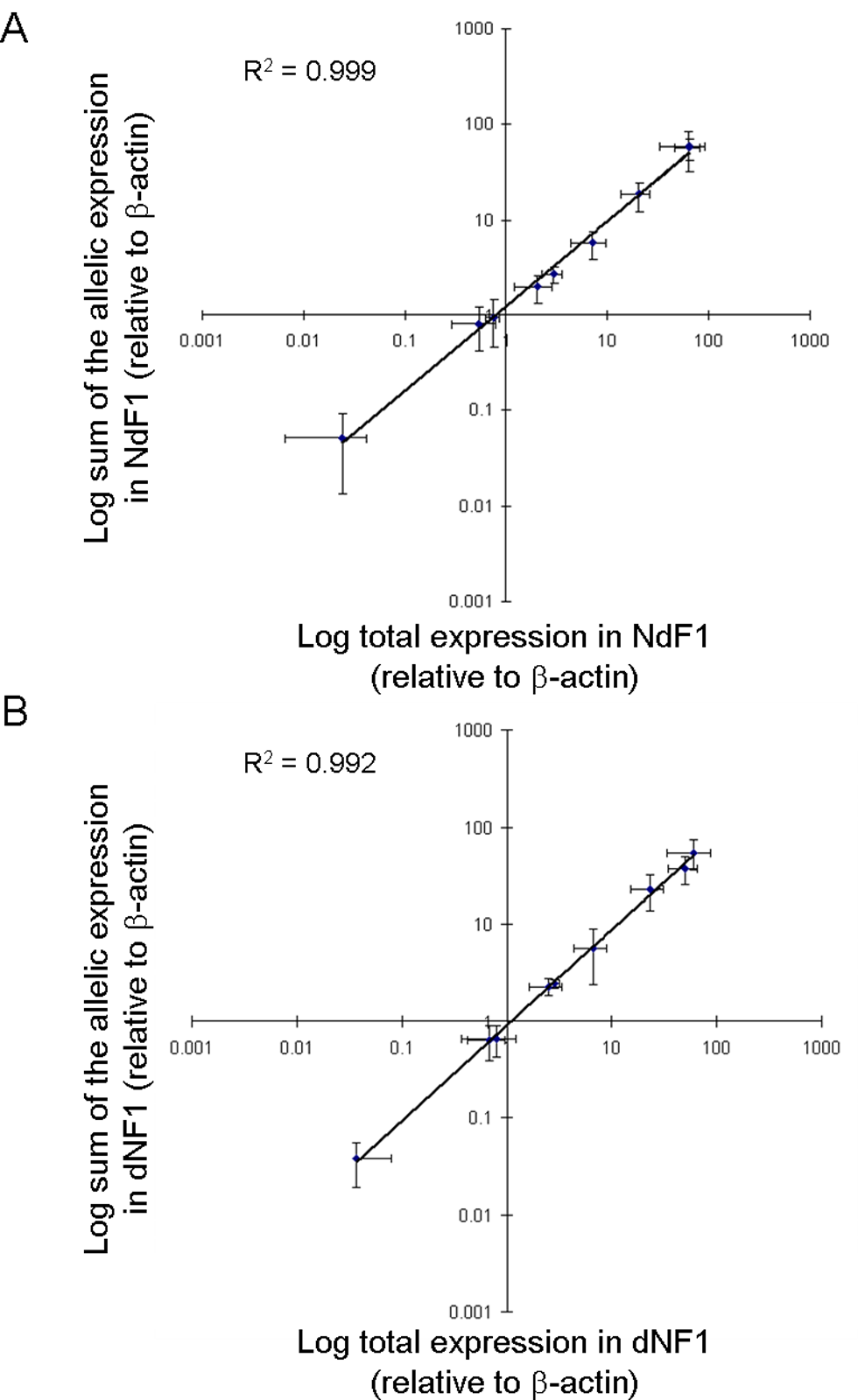

Supplement: Figure S4 — Comparison of total expressions and sum of the two allele expressions of three genes (MCM2, HPRT1 and CYP2J2) in intestine, liver and brain of reciprocal hybrids. Total expression and sum of the two allele expressions of 11 genes in intestines of NdF1 (A) and dNF1 (B) were quantified by common primers and the two allele-specific primers, respectively. All expressions were normalized by β-actin expressions and were plotted on a logarithmic scale. (TIF) [file pone.0036875.s004.tif]

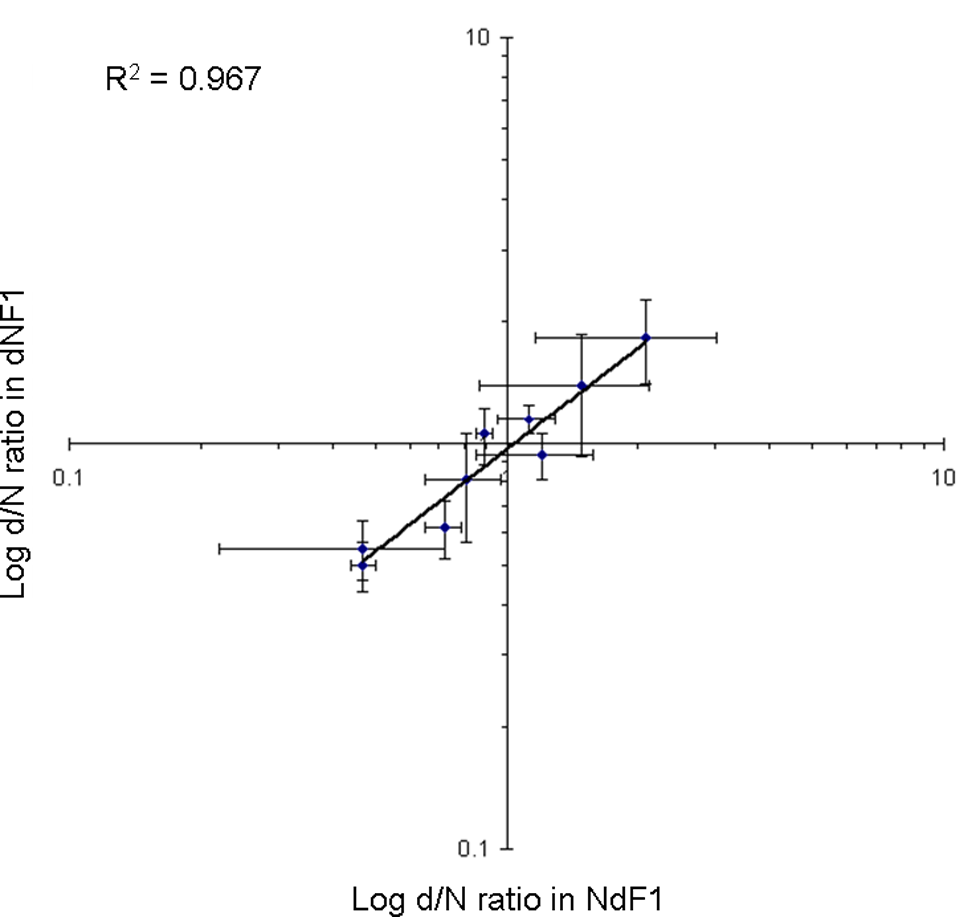

Supplement: Figure S5 — Comparison of the d/N ratios of 3 genes (MCM2, HPRT1 and CYP2J2) in intestine, liver and brain of reciprocal hybrids. All d/N ratio were plotted on a logarithmic scale. (TIF) [file pone.0036875.s005.tif]

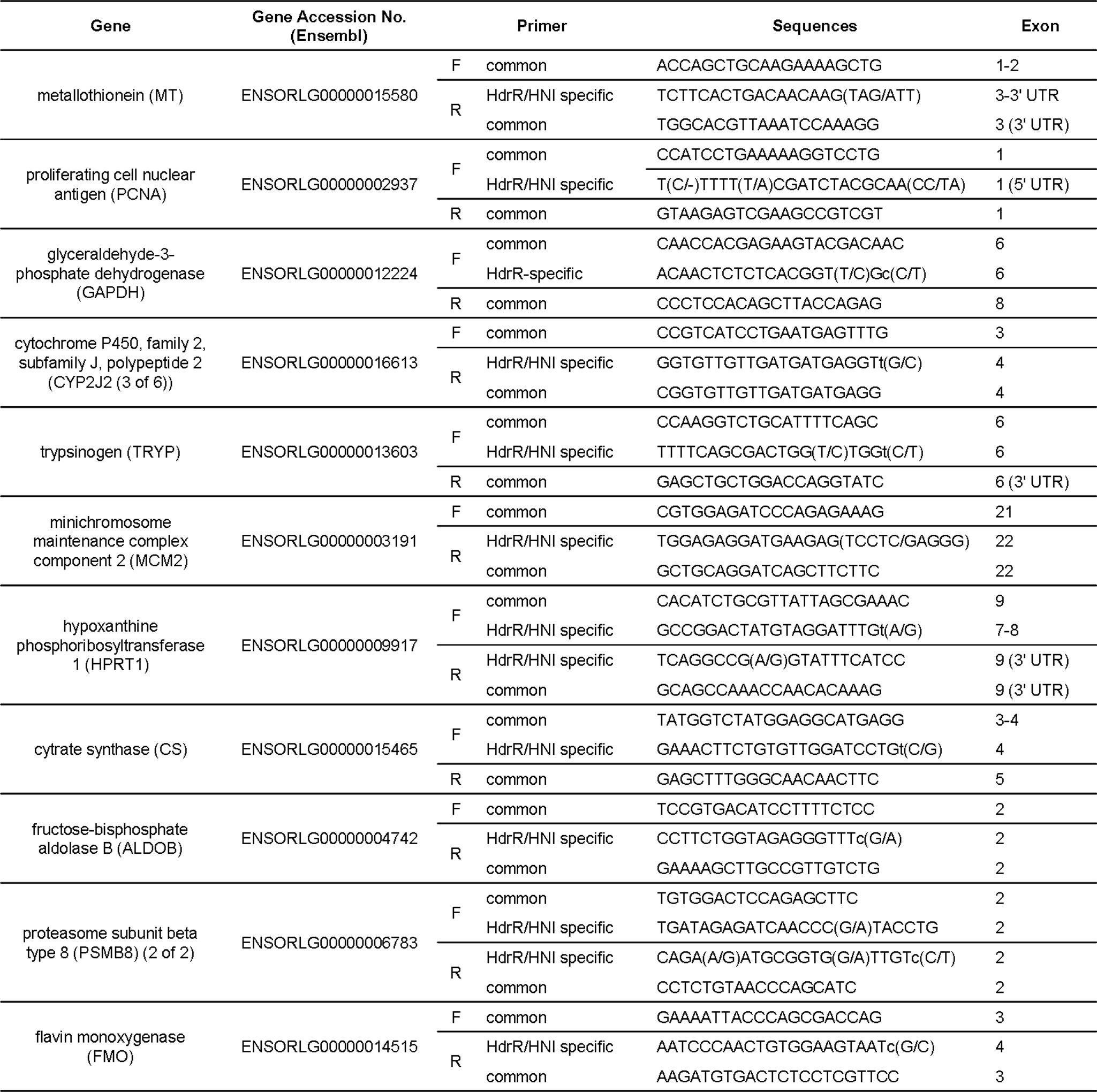

Supplement: Table S1 — All common and allele-specific primer sequences and locations for the 11 genes. Small letters in primer sequences represent mismatch nucleotide. (TIF) [file pone.0036875.s006.tif]

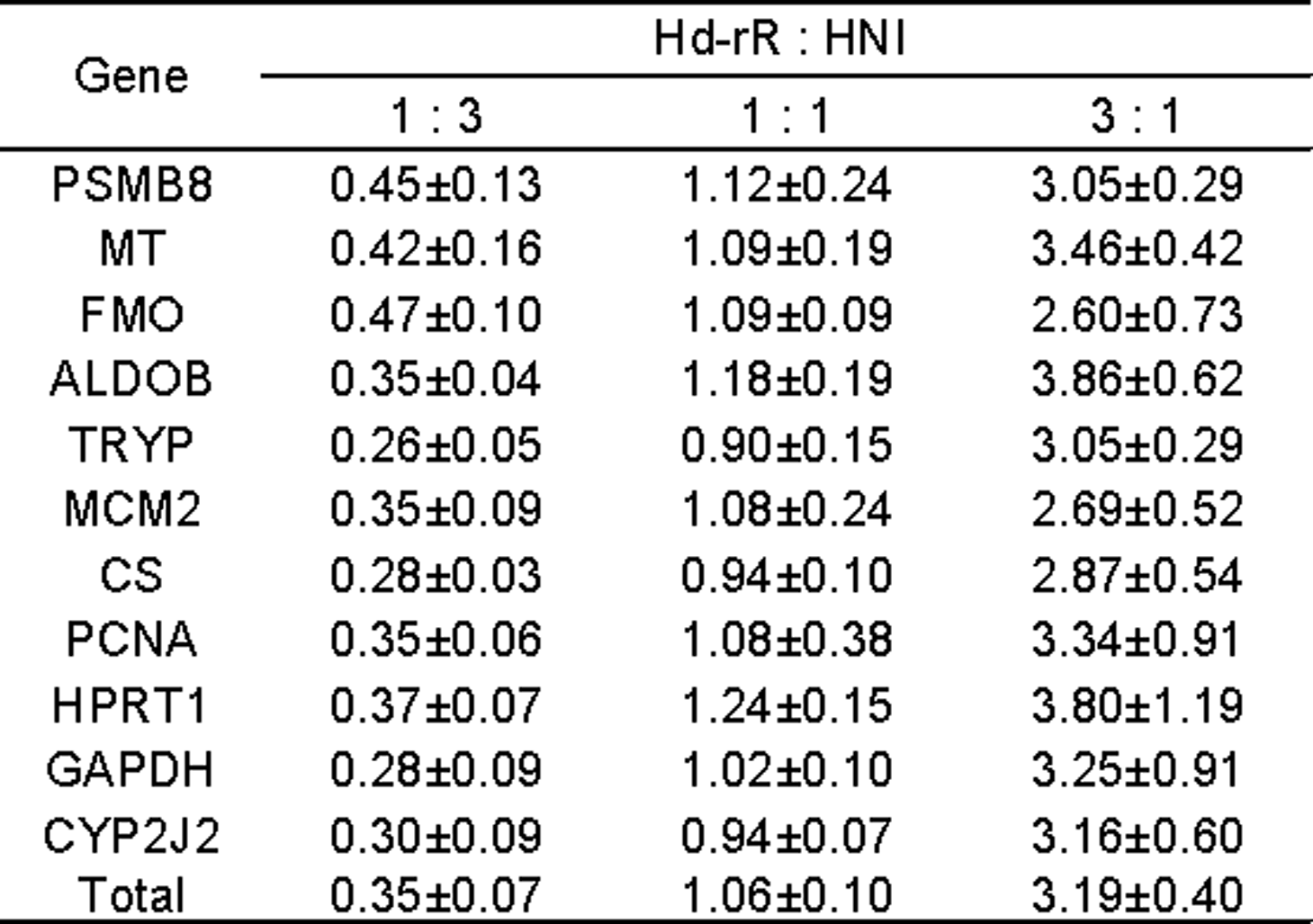

Supplement: Table S2 — Hd-rR and HNI allele expression ratio of 11 genes were quantified using a mixed cDNA of parental strains at known ratio (HNI allele expression∶Hd-rR allele expression; 1∶3, 1∶1 and 3∶1). All expressions were normalized by β-actin expressions. Data is presented as mean ± SD, n = 3. (TIF) [file pone.0036875.s007.tif]

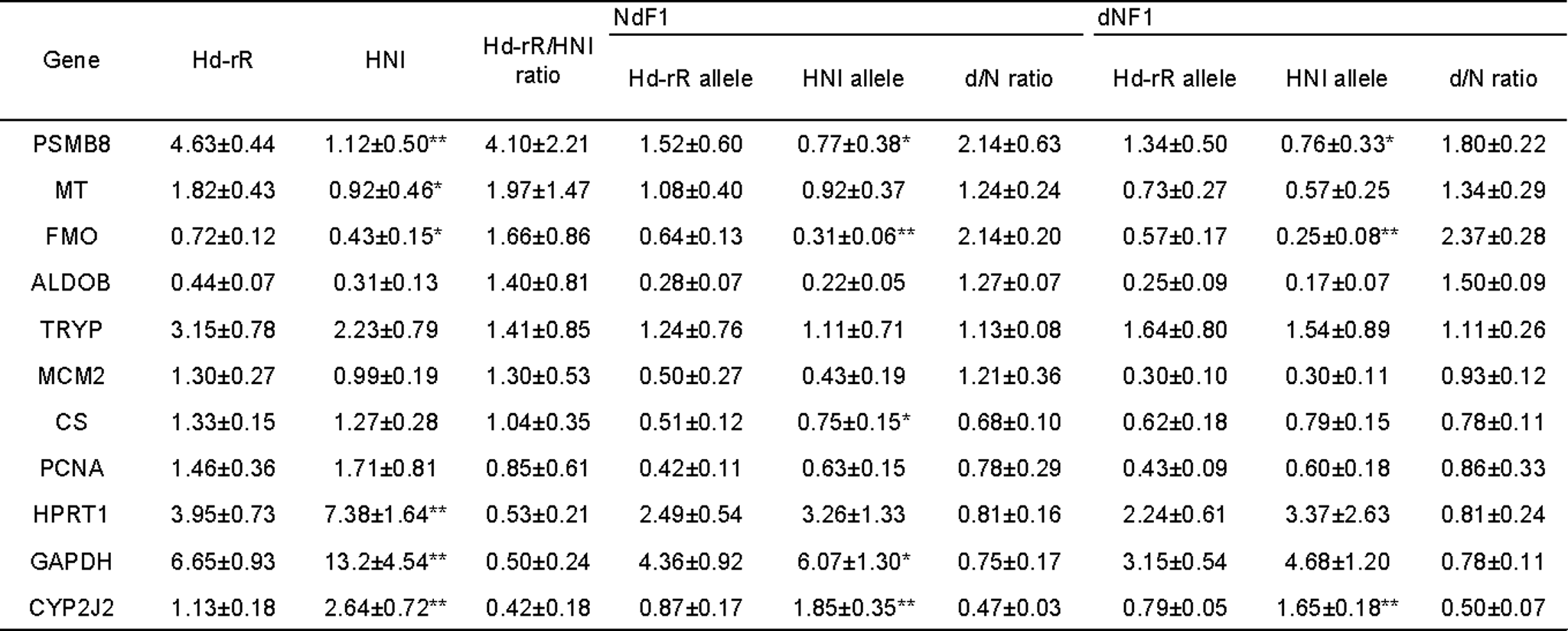

Supplement: Table S3 — Total expressions of 11 genes in parental strains and allelic expression of 11 genes in intestines of reciprocal hybrids quantified by common and allele-specific primers. Hd-rR/HNI ratio: (total expression in Hd-rR)/(total expression in HNI), d/N ratio: (Hd-rR allele expression)/(HNI allele expression). NdF1: hybrid of female HNI and male Hd-rR, dNF1: hybrid of female Hd-rR and male HNI. All expressions were normalized by β-actin expressions. Data is presented as mean ± SD, n = 6. *P<0.05, **P<0.01. (TIF) [file pone.0036875.s008.tif]

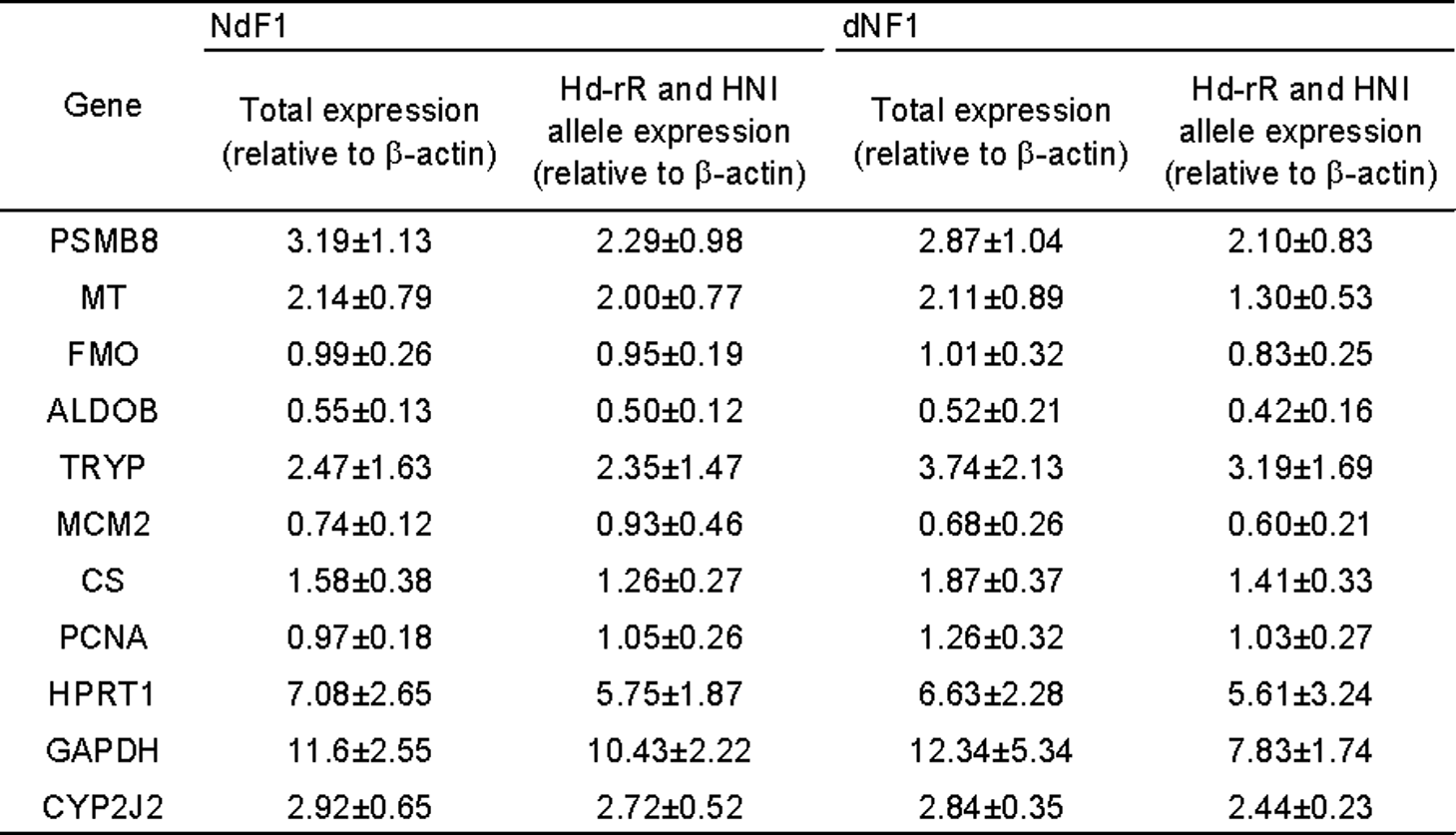

Supplement: Table S4 — Total expression of 11 genes and sum of the two allele expressions of 11 genes in intestines of reciprocal hybrids quantified by common and allele-specific primers. All expressions were normalized by β-actin expressions. Data is presented as mean ± SD, n = 6. (TIF) [file pone.0036875.s009.tif]

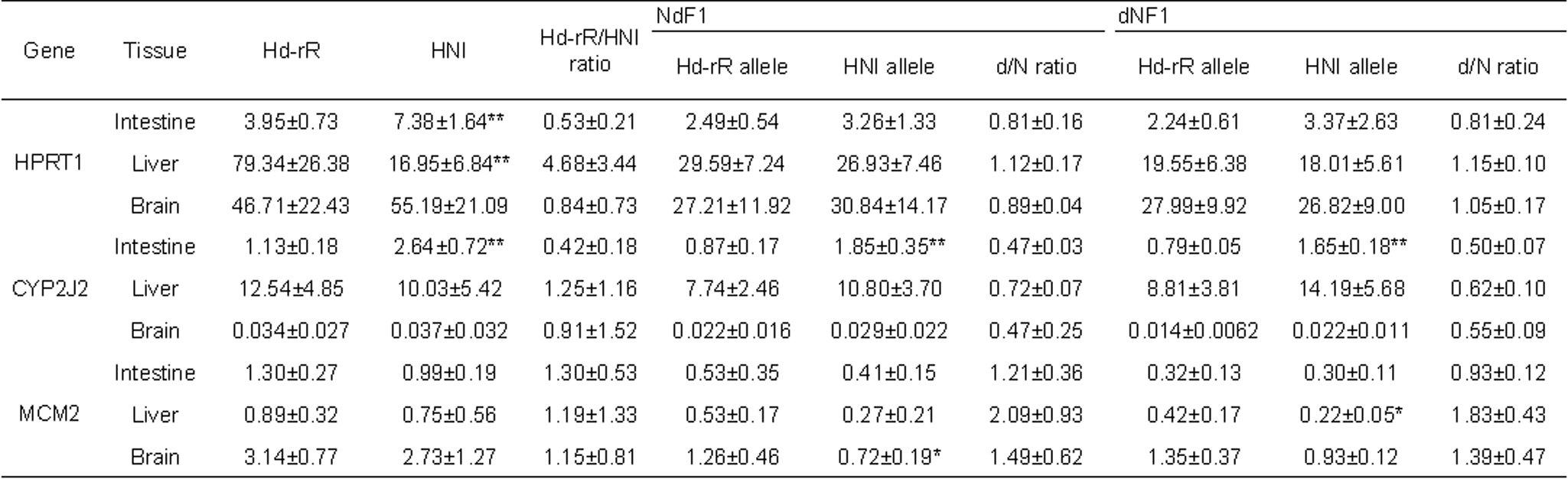

Supplement: Table S5 — Total expressions of 3 genes (HPRT1, CYP2J2 and MCM2) in parental strains and allelic expression of 3 genes in three tissues (intestine, liver and brain) of reciprocal hybrids quantified by common and allele-specific primers. Hd-rR/HNI ratio: (total expression in Hd-rR)/(total expression in HNI), d/N ratio: (Hd-rR allele expression)/(HNI allele expression). NdF1: hybrid of female HNI and male Hd-rR, dNF1: hybrid of female Hd-rR and male HNI. All expressions were normalized by β-actin expressions. Data is presented mean ± SD, n = 6 *P<0.05, **P<0.01. (TIF) [file pone.0036875.s010.tif]

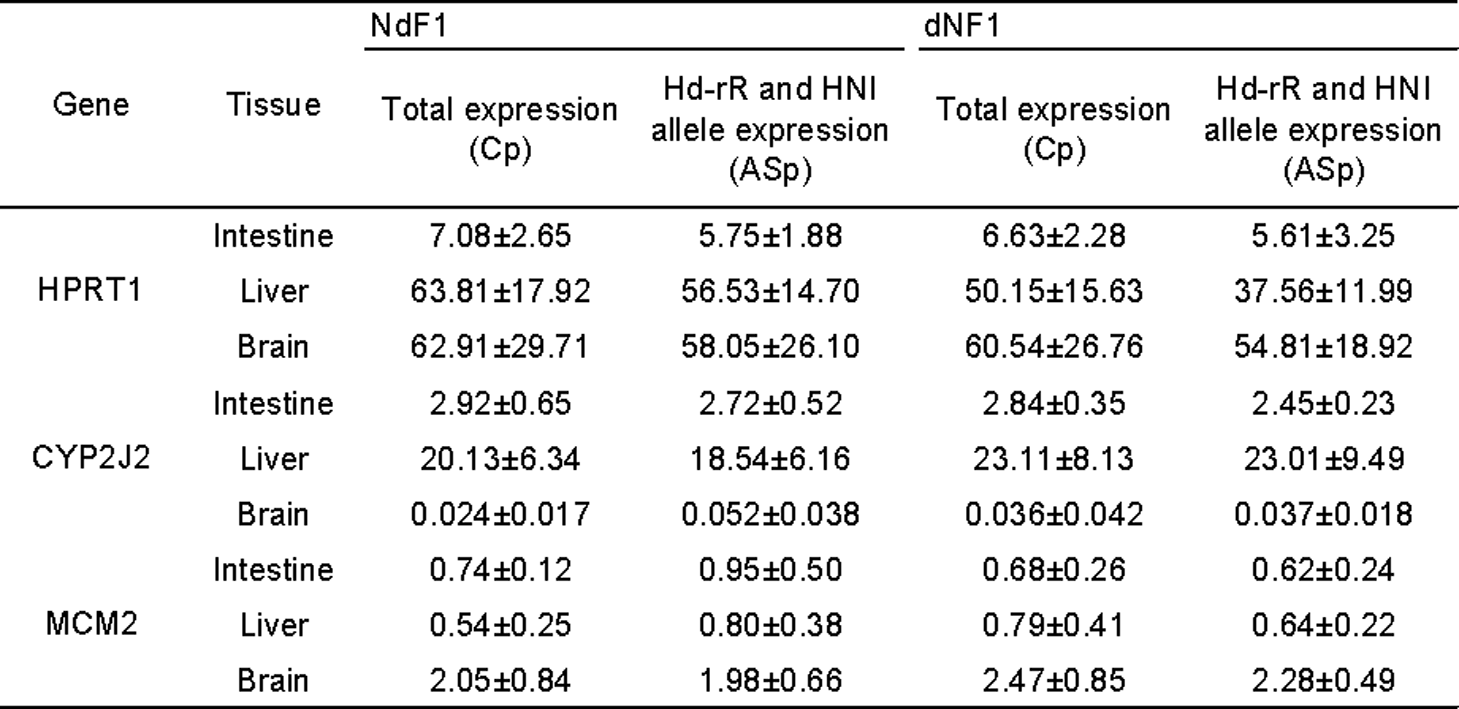

Supplement: Table S6 — Total expression of 3 genes (HPRT1, CYP2J2 and MCM2) and sum of the two allele expressions of 3 genes in three tissues (intestine, liver and brain) of reciprocal hybrids quantified by common and allele-specific primers. All expressions were normalized by β-actin expressions. Data is presented as mean ± SD, n = 6. (TIF) [file pone.0036875.s011.tif]
